# Single-Nucleotide Polymorphism-Based Genetic Diversity Analysis of Clinical *Pseudomonas aeruginosa* Isolates

**DOI:** 10.1093/gbe/evaa059

**Published:** 2020-04-01

**Authors:** Uthayakumar Muthukumarasamy, Matthias Preusse, Adrian Kordes, Michal Koska, Monika Schniederjans, Ariane Khaledi, Susanne Häussler

**Affiliations:** e1 Department of Molecular Bacteriology, Helmholtz Centre for Infection Research, Braunschweig, Germany; e2 Institute of Molecular Bacteriology, TWINCORE GmbH, Center for Clinical and Experimental Infection Research, Hannover, Germany

**Keywords:** pan-genome, core genome, SNPs, convergent and divergent evolution

## Abstract

Extensive use of next-generation sequencing has the potential to transform our knowledge on how genomic variation within bacterial species impacts phenotypic versatility. Because different environments have unique selection pressures, they drive divergent evolution. However, there is also parallel or convergent evolution of traits in independent bacterial isolates inhabiting similar environments. The application of tools to describe population-wide genomic diversity provides an opportunity to measure the predictability of genetic changes underlying adaptation. Here, we describe patterns of sequence variations in the core genome among 99 individual *Pseudomonas aeruginosa* clinical isolates and identified single-nucleotide polymorphisms that are the basis for branching of the phylogenetic tree. We also identified single-nucleotide polymorphisms that were acquired independently, in separate lineages, and not through inheritance from a common ancestor. Although our results demonstrate that the *Pseudomonas aeruginosa* core genome is highly conserved and in general, not subject to adaptive evolution, instances of parallel evolution will provide an opportunity to uncover genetic changes that underlie phenotypic diversity.

## Introduction

A major challenge in biological and environmental research is to understand the intricate link between genotypes and phenotypes and how the environment influences that link. Genetic variants such as mutations or the presence or absence of a gene among individuals can produce different phenotypes that undergo positive or negative selection. Thus, evolutionary change critically relies on genetic variations. The genetic variation within a species is often called genetic diversity and typically corresponds to phenotypic variation. In a genetically diverse population, it is more likely that at least some individuals survive in changing environments (Sokurenko et al. 1999; Ziebuhr et al. 1999).

A promising approach to understand the evolution of genetic diversity is to study genetic changes within populations as they adapt to novel and challenging habitats. Habitats that are suboptimal for sustained growth provide strong selection for adaptive changes. The opportunistic human pathogen *Pseudomonas aeruginosa* represents an excellent model for understanding the molecular mechanisms of the adaptation of a bacterial species to a variety of challenging habitats. The species *P. aeruginosa* inhabits a stable source habitat in the natural environment, which serves as a reservoir to cause infectious diseases in humans occasionally. If not eradicated during acute infection, *P. aeruginosa* populations can cause chronic infections that exist for a long enough time for evolution to take place (Sokurenko et al. 2006; Yang et al. 2011).

Based on annotated genes across a set of 99 genotypic diverse clinical *P. aeruginosa* isolates, we used established BLAST-based alignment tools to construct a pan-genome, which provided a robust framework for the detection of the genetic diversity within the 3,814 core genes of our clinical *P. aeruginosa* strain collection at the single-nucleotide level. We were able to identify and define sequence variations associated with certain phylogroups (divergent signatures). Our analysis also uncovered convergent adaptation signatures that evolved independently of the phylogenetic background of the clinical isolates. Analysis of the strict core genes of a collection of 99 clinical isolates furthermore revealed that the *P. aeruginosa* core genome is highly conserved across clinical isolates and is generally not subject to adaptive evolution.

## Materials and Methods

### Bacterial Strains and Genomic Sequencing

In this study, we sequenced the genomes of 99 clinical isolates of *P. aeruginosa* that were sampled from various infection sites at different hospitals across Germany. Only one isolate per patient was included in this study. Genomic DNA was prepared from *P. aeruginosa* isolates using the NEBNext Ultra Kit and sequenced on an Illumina MiSeq System, generating 2× 300-bp paired-end reads. Using a multiplexed protocol, an average of 1,037,171 reads (range of 512,812–1,645,685) for each of the genomic libraries were obtained. On average, the isolates were sequenced with an estimated genomic coverage of 68-fold (supplementary file 1, Supplementary Material online).

### De Novo Assembly, Annotation, and Generation of the Pan-Genome

Preprocessing, such as the removal of adapter and bar code sequences, was done using the FASTQ-MCF script provided by EA-UTILS (https://expressionanalysis.github.io/ea-utils/, last accessed March 4, 2020) (Aronesty 2013); Karect was used for error correction (Allam et al. 2015). The processed reads were assembled de novo using the A5-miseq pipeline (Coil et al. 2015). Its built-in scaffolder, SSPACE v3 (Boetzer et al. 2011), was used to generate scaffolds from the assembled contigs resulting in an average of 40 scaffolds per isolate (13–192). Final assembled scaffolds were parsed to generate gene annotations using Prokka v1.11 (Seemann 2014).

**Fig. 1.**
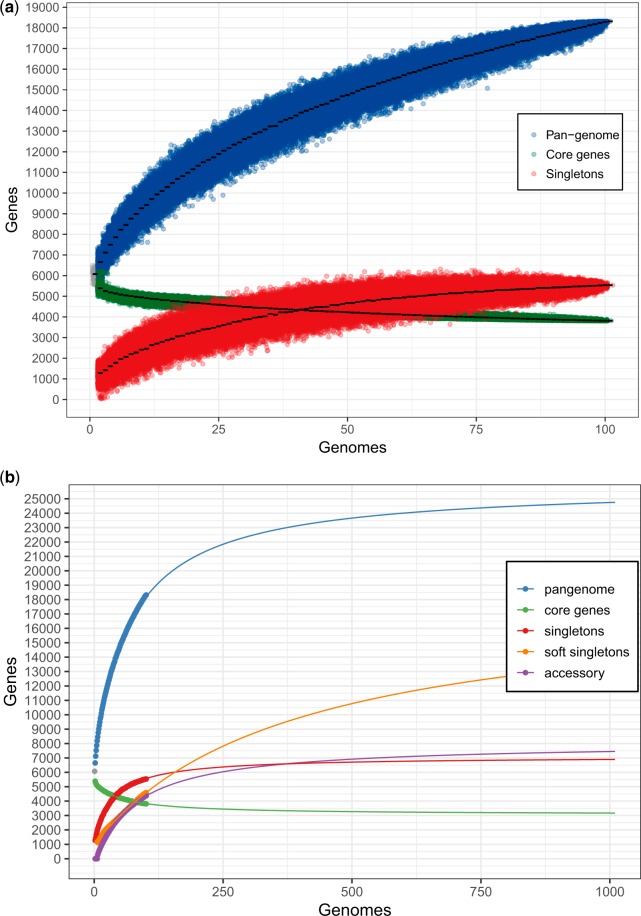
—Influence of the number of sequenced genomes on the *P. aeruginosa* pan- and core-genome size. (*A*) The number of genes belonging to the pan-genome (blue dots), core genome (green dots), and singletons (red dots) is plotted as a function of genomes sequentially added in all possible combinations (limited to 1,000). (*B*) Saturation model of for the pan (blue), core (green), singleton (red), soft singleton (genes present in only 2–5% of the isolates, orange), and accessory (purple) genomes. On 101 genomes, the model predicts a pan-genome saturation of 95% with 940 genomes that correspond to a pan-genome size of 25,946 genes.

The standard reference genomes of *P. aeruginosa* PA14 and PAO1 strain types were downloaded from GenBank (Benson et al. 2018) and added to the current data set. With the aim to assign genes to orthologous groups, all gene sequences were blasted against each other using BlastN (Altschul et al. 1990), discarding hits having <90% length and 90% sequence identity. Only genes having reciprocal homologs in all 101 genomes were considered as **“**core genome.” Genes that were present in only a fraction of genomes were considered **“**accessory genome.” A gene presence/absence matrix was used for determining how the number of singleton, pan-, and core-genome genes develops with an increasing number of genomes *x* in the data set. One thousand out of the possible combinations were sampled for each number of genomes *x* to generate figure 1*A*. Next, the further development of singletons, pan-, and core-genome beyond the 101 genomes used here was predicted by fitting a saturation model, *y* = *c* ± [*a* × *x*/(*b* + *x*)], to the data (fig. 1*B*). The letters *x* and *y* indicate the number of genomes and genes, respectively. The letters *a* and *b* affect arc height and slope of the saturation curve, respectively. The model was optimized using the Nelder–Mead method which is part of the R function **“**optim” (Nelder and Mead 1965).

### Core Genome Multilocus Sequence Typing

A phylogenetic tree was constructed based on sequence variations within the 3,814 core genes. All core genes were concatenated, resulting in one fasta sequence per isolate. Phylogenetic distances between the strains were calculated using a *k*-mer approach as described previously (Leekitcharoenphon et al. 2014). The sequences were split into 15-mers (and into 22-mers to construct the phylogenetic tree of 99 isolates plus all 52 reference genomes) which were then compared between the isolates. The resulting distance matrix was used to build a Neighbor-Joining tree in R using the ape package (Paradis et al. 2004). SRST tool and BIGSdb database were used to identify the sequence type (ST) information for each isolate (Jolley and Maiden 2010; Inouye et al. 2014). This information was supplemented and visualized as a phylogenetic tree using iTOL (Letunic and Bork 2016) and midpoint rooted. Also, an approximately-maximum-likelihood phylogenetic tree was constructed using fasttree 2.1.10 with generalized time-reversible model (Price et al. 2010).

### Single-Nucleotide Polymorphism Identification in the Core Genes

**Fig. 2.**
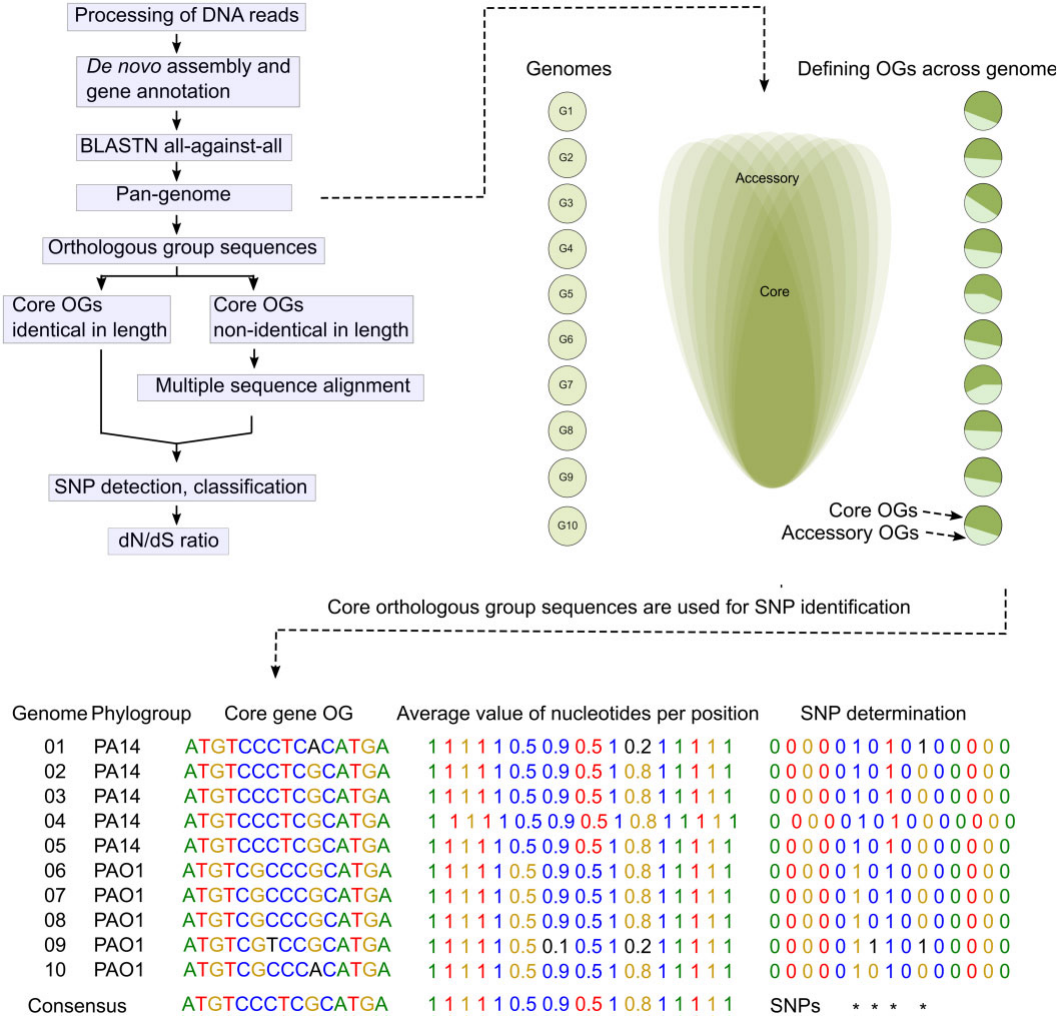
—Framework for SNP identification in the core genes. Raw DNA sequencing reads are processed and error corrected before de novo assembly and gene annotation. Orthologous groups (OGs) are identified based on an all-against-all sequence comparison of the genes in the assembled genomes. Each core gene orthologous group is aligned. The average value of each type of nucleotide at each position is calculated. Values are converted into 0s and 1s according to the most occurrences and least occurrences, respectively, and 1s are determined as sequence variations. Phylogroup information is further used to classify the divergent and convergent adaptation patterns.

The gene sequences were extracted from the corresponding genomes for every core gene orthologous group. If genes within one orthologous group were identical in length, the orthologous sequences were directly aligned. If the genes within an orthologous group were nonidentical in length, a multiple sequence alignment was performed by using clustal-omega (Sievers et al. 2011). Sequence variations at each nucleotide position in all core genes across the 101 genomes were recorded. Phylogroup information was further used to classify the divergent and convergent adaptation patterns. (See figure 2 for the framework.)

### d*N*/d*S* Ratio

The ratio of the rate of nonsynonymous substitutions (d*N*) to the rate of synonymous substitutions (d*S*) was calculated based on the modified Nei and Gojobori method (Nei and Gojobori 1986) through the use of SNAP (Korber 2000). A total of 3,814 gene sequences from each isolate were used to calculate the d*N*/d*S* ratio in a pairwise manner.

### Data Availability

All short-read data are available at the National Center for Biotechnology Information Sequence Read Archive (http://www.ncbi.nlm.nih.gov/sra, last accessed March 4, 2020) under accession number SRP160898.

## Results

### The *P. aeruginosa* Pan-Genome

Whole-genome sequencing of a multitude of bacterial isolates from one species gives detailed information on the available gene pool. This approach provides information on the number of genes that are common to all isolates (core genome), the number of genes that are found in a subset of strains (accessory genome), and those that only occur in a single strain (singletons) (Medini et al. 2005; Tettelin et al. 2005). However, whole-genome sequencing also gives information on the sequence variation on the single-nucleotide level. In this study, we sequenced the genomes of 99 clinical *P. aeruginosa* isolates with an Illumina MiSeq System. Clinical isolates were obtained from numerous infection sites and patients across Germany (supplementary file 1, Supplementary Material online). To generate the pan-genome, we performed a de novo assembly of the aforementioned genomes. The fully sequenced two *P. aeruginosa* reference strains, PA14 and PAO1, were included in the generation of the pan-genome. De novo annotation revealed an average gene content of 6,255 genes (5,677–6,831) per individual isolate (details in supplementary file 1, Supplementary Material online). We collapsed the genes into orthologous groups by blasting all-against-all using BlastN. If a gene had a reciprocal set of orthologs across all strains, the corresponding gene was considered core; otherwise it was considered accessory. This produced 18,319 nonredundant genes between the 101 strains; 14,505 of these genes were categorized as accessory (5,539 of which were singletons), and 3,814 were characterized as core. Of note, a large number of genes within the accessory genome (1,257) were present in almost all strains (95–99% of all isolates) (supplementary fig. S1, Supplementary Material online). Closer inspection revealed that many of these genes were not completely absent between isolates but were affected by partial gene losses (also due to artifacts from the assembly). Therefore, we classified these 1,257 genes as soft-core genes. However, among those soft-core genes, there might be true accessory genes, as some genes might be completely absent in a small fraction of isolates (<5%). There may also be additional soft-core genes. For example, *lasR* exhibited (incomplete) gene losses in more than 5% of our clinical isolates, and therefore was not considered as soft core, but as an accessory gene in our analysis. Thus, our total of 1,257 soft-core genes is only an estimate that could be further curated as more isolates are sequenced.

The main features of our 99 isolate-derived *P. aeruginosa* pan-genome were comparable to those previously published. We found a large number of strict (3,814) and soft (1,257) core genes. This was in line with previous reports on a high degree of conservation between *P. aeruginosa* isolates (Mathee et al. 2008; Klockgether et al. 2011; Hilker et al. 2015; Valot et al. 2015). The size of the pan-genome increased steadily with the addition of each further genome (fig. 1*A*). The higher expansion rate for the pan-genome size in comparison to that of the singletons may suggest that selection is involved in the expansion of the pan-genomes and that newly acquired advantageous genes quickly expand along the tree, whereas others are purged.

However, clonal lineages within the *P. aeruginosa* population might bias the pan-genome evolution as a clonal structure makes it more and more unlikely that new singletons are found, if the population increases. We therefore generated a saturation model and defined *soft-singleton* genes, which are present in only few (2–5) isolates. It was estimated that the pan-genome is saturated with 25,946 genes and 940 genomes would provide a 95% saturation. Saturation was also estimated with 3,050 strict core genes (683 genomes for 95% saturation), 7,076 singleton genes and 18,475 soft singletons (536 genomes and 6949 genomes for 95% saturation, respectively). Furthermore, a saturated accessory genome size of 8,061 genes was estimated (6,812 genomes would provide a 95% saturation) (fig. 1*B*). The finding that the expansion rates for the pan-genomic and the genes that are present in only few (2–5) isolates (soft singletons) exhibited a close to parallel evolution indicates that the *P. aeruginosa* pan-genome evolved neutrally.

### Phylogenetic Relationship of the Clinical *P. aeruginosa* Isolates

**Fig. 3.**
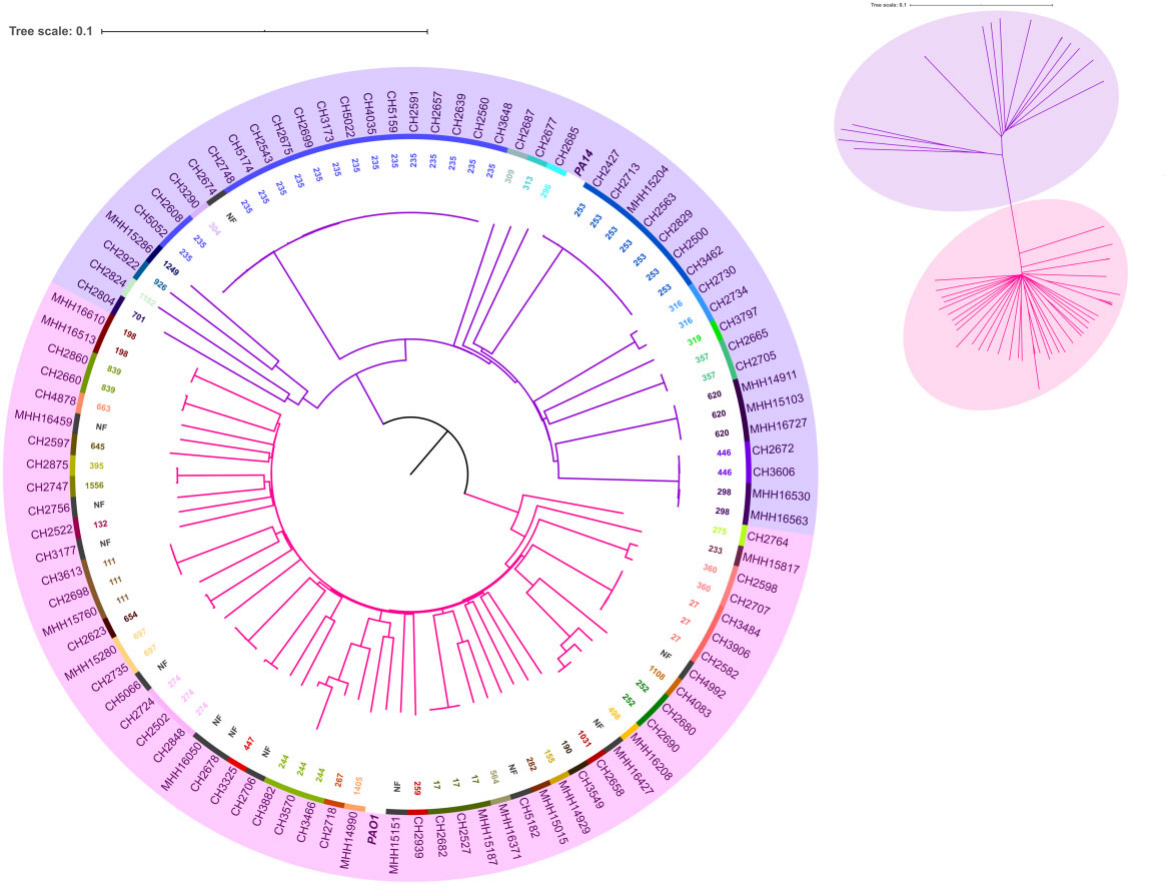
—Broad phylogenetic distribution of the 99 *P. aeruginosa* clinical isolates. The equal-angle unrooted tree on the top right indicates that the isolates separate into two clonal complexes. The midpoint rooted circular tree highlights in pink the PAO1-like isolates and in violet the PA14-like isolates. Strain types PAO1 and PA14 highlighted in bold. ST information is shown as an inner ring at different color scale. NF indicates newly identified STs that are not found in the database.

We constructed a Neighbor-Joining tree with our 99 clinical isolates based on a distance matrix calculated from *k*-mers of the 3,814 core genes. The core genome, which includes the seven standard multilocus sequence type (MLST) genes of *P. aeruginosa* (*aroE*, *trpE*, *guaA*, *nuoD*, *ppsA*, *acsA*, and *mutL*) (Curran et al. 2004), represents more than 61% of the average *P. aeruginosa* genome size, thereby providing a fine-scale resolution of clonal linages. This phylogenetic tree largely consists of two major nonoverlapping clusters, containing the PA14 and the PAO1 type strains, respectively (Wiehlmann et al. 2007; Freschi et al. 2015) (fig. 3). Forty-four clinical *P. aeruginosa* isolates clustered with the PA14 phylogroup, whereas the remaining 55 isolates formed a PAO1 phylogroup. The tree also provides information on more than 40 distinct STs, including major STs ST235, ST111, and ST132. These subgroups were identified based on the MLST profiles of *P. aeruginosa*. The phylogenetic distribution of our 99 clinical isolates was found to be comparable to the phylogenetic diversity of 52 previously sequenced *P. aeruginosa* strains (supplementary fig. S2 and file 1, Supplementary Material online).

### Alignment of the *P. aeruginosa* Core Genes

Among the 3,814 core genes, 3,014 genes were identical in length within their orthologous groups. For the remaining 800 genes that were nonidentical in length to their corresponding group, a standard multiple sequence alignment was performed to identify the gaps. The orthologous groups served as the basis for the generation of a quality-rich reference structure of the respective core gene. A visualization of the genetic diversity at the single-nucleotide level of *P. aeruginosa* core genes is provided in the Bactome database (https://bactome.helmholtz-hzi.de, last accessed March 4, 2020) (Hornischer et al. 2019) and exemplarily in supplementary figure S3, Supplementary Material online.

### Identification of Single-Nucleotide Polymorphisms in the *P. aeruginosa* Phylogroups

The 3,814 core genes (corresponding to 3,629,979 nucleotide positions) were scanned for sequence variations across the 99 *P. aeruginosa* genomes amounting to overall 159,609 single-nucleotide polymorphisms (SNPs). This corresponds to a mean sequence diversity at the single-nucleotide level of 0.04 (number of SNP positions/total number of nucleotide positions), indicating that the core genes are highly conserved within these 99 strains (Spencer et al. 2003; Wiehlmann et al. 2007; Dötsch et al. 2010). Seven genes—namely, *rpsS*, *rpmC*, *minE*, *acpP*, *lppL*, PA14_07370, and PA14_12560—did not harbor any SNPs across the isolates. We found 49,722 SNPs which were isolate-specific with 27,911 of them were found exclusively in the PA14 phylogroup, whereas the remaining 21,811 SNPs were found in isolates belonging to the PAO1 phylogroup.

**Fig. 4.**
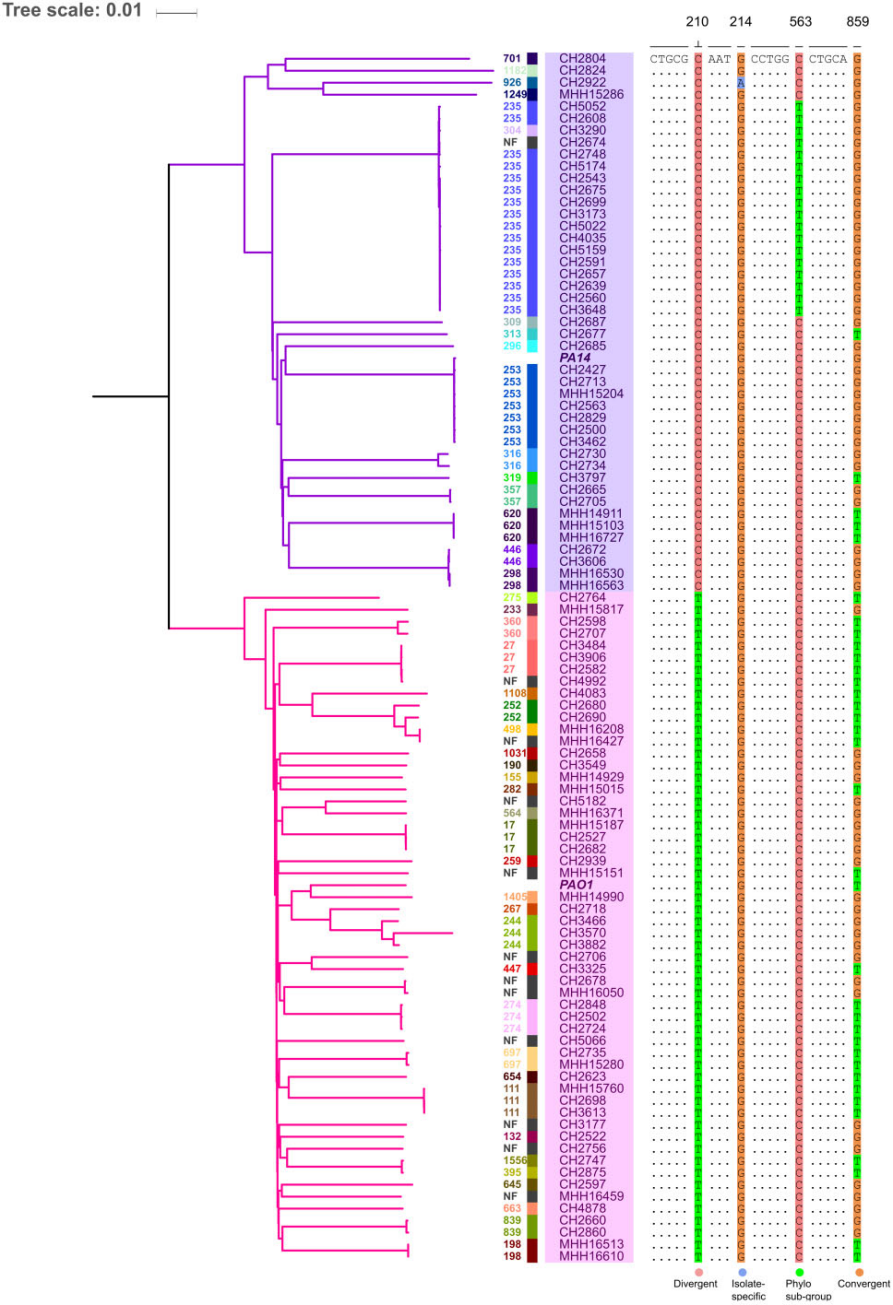
—Classification of SNPs across isolates. The rooted phylogenetic tree (on the left) reflects the ST-specific information in two major groups. On the right, four nucleotide positions 210, 214, 563, and 859 of PA14_00190 serve as an example to demonstrate the different classes of SNPs. At position 210, the SNP is 100% divergent; at position 214, there is an isolate-specific SNP, that is, present only in isolate-CH2922; at position 563, a phylogenetic subgroup specific SNP present in PA14 phylogroup and absent in PAO1 phylogroup; and at position 859, the SNP is commonly identified in the clinical isolates and is found independent on the phylogenetic background (convergent).

As expected, the sequence variation among the isolates was not random, but isolates that belonged to the same phylogroup shared patterns of SNP profiles (fig. 4). The PA14 and PAO1 clonal complexes have diverged evolutionary (Lee et al. 2006), and we identified 463 positions where the entire 44 PA14-like isolates could be distinguished from the 55 PAO1-like isolates (strict phylogroup-associated SNPs: present in all isolates of one [PAO1 or PA14] phylogroup and in none of the other phylogroup). In total, 8,410 SNPs were present in all isolates of a single phylogroup, with one or two isolates of the other group harboring the same SNP. In addition to these phylogroup-SNPs, 66,892 SNPs were found to be exclusively present in phylogenetically closely related isolates. These SNPs were typically present in only one phylogenetic subgroup, but absent outside the group. In other words, SNPs were found in <44 of the PA14-like isolates and completely absent in all 55 PAO1-like isolates; or SNPs were found in <55 of the PAO1-like isolates and completely absent in all 44 PA14-like isolates. Overall, 75,765 phylogroup-associated (divergent) SNPs were classified by our approach; the majority of which did not result in a change in the amino acid sequence of the encoding protein.

**Fig. 5.**
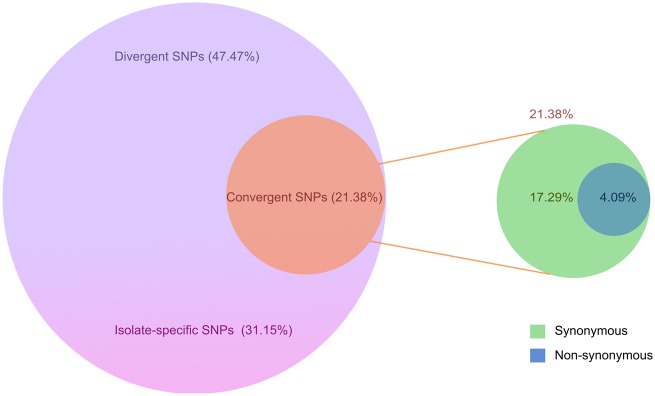
—Various sets of SNPs within the core genome of *P. aeruginosa*. In a total of 159,609 SNPs, ∼47.47% are divergent—highlighted in violet; 31.15% are isolate-specific—highlighted in pink; and 21.38% are identified in the isolates independent on the phylogenetic background (convergent)—highlighted in orange. The latter SNPs include 17% of synonymous mutations—highlighted in green and 4% of nonsynonymous mutations—highlighted in blue.

In addition to the 75,765 phylogroup/divergent SNPs and the 49,722 single SNPs (which occurred only in one isolate), a total of 34,122 SNPs were identified across many isolates, independent of the phylogenetic background (21.38% of the total SNPs in 3,567 genes). Of these 34,122 SNPs, 27,590 SNPs (∼80%) were identified as synonymous, whereas 6,532 (∼ 20%) were identified as nonsynonymous. Those SNPs were found in 3,497 and 2,252 genes, respectively (fig. 5). A list that ranks these 2,252 genes according to the relative frequency of nonsynonymous mutations and normalized to gene length is provided in supplementary file 1, Supplementary Material online. Also, the frequency of mutations at identical nucleotide positions across the clinical isolates was determined. The position-wise mutations were sorted based on the number of occurrences across the total number of isolates and are presented in supplementary file 1, Supplementary Material online.

We used the 75,765 SNPs that are divergent in the population to construct a phylogenetic tree. In comparison to the original tree based on all core genome SNPs, the tree shows a slight variation in distances across and within phylogroups and depicts clear sorting within phylogroup nodes, especially in PA14 group (supplementary fig. S4, Supplementary Material online).

### d*N*/d*S* Ratio as a Measure of Selective Pressure

**Fig. 6.**
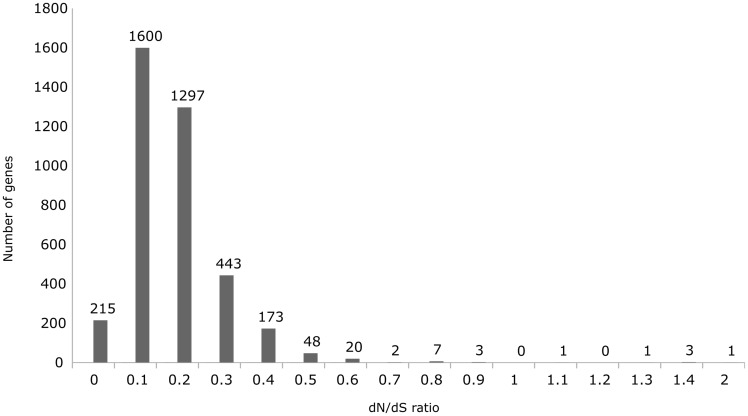
—d*N*/d*S* ratio (omega values) for the overall 3,814 core genes. The mean pairwise d*N*/d*S* ratio for the 3,814 core genes is 0.14. The ratio of almost all the core genes is between 0 and 1. There are only six genes that show the d*N*/d*S* ratio between 1 and 2.

The ratio of nonsynonymous substitutions rates (d*N*) to synonymous substitutions rates (d*S*), d*N*/d*S*, remains one of the most common measures used to describe stabilizing selection. A ratio of d*N*/d*S* > 1 indicates positive selection, a d*N*/d*S* ratio of 1 corresponds to neutral selection, and d*N*/d*S* < 1 denotes a purifying (or negative) selection (Yang and Bielawski 2000; Rocha et al. 2006). To determine the synonymous and nonsynonymous substitution rates and the selective evolutionary pressure, a pairwise comparison approach was applied on the core genes using the SNAP program based on the NG86 method (Korber 2000). The mean pairwise d*N*/d*S* ratio for the 3,814 core genes was found to be 0.14 (fig. 6). This suggests that the core genome of *P. aeruginosa* is under purifying selection as a whole, which is in agreement with previous analyses (Yang et al. 2011; Mosquera-Rendón et al. 2016). Only six core genes (*bfrG*, *fptB*, *napA*, PA14_11160, PA14_65950, and PA14_69250) exhibited a d*N*/d*S* ratio between 1 and 2. In an extended analysis, we determined the d*N*/d*S* ratio for the genes present in 2–5 isolates (4,362 soft singletons). The d*N*/d*S* ratio of 0.06 indicates that there is no positive selection on these genes (fig. 1*B* and supplementary fig. S5, Supplementary Material online).

## Discussion

Advances in next-generation sequencing have opened up new frontiers in microbial genomics. Relatively inexpensive NGS technologies have the ability to produce large quantities of sequencing data, providing the opportunity to compare sequence variation between two or more strains, and enabling a detailed analysis of the genetic variation within a species (Oliver et al. 2000; Smith et al. 2006; Bragonzi et al. 2009; Cramer et al. 2011; Yang et al. 2011; Folkesson et al. 2012; Marvig et al. 2015; Mosquera-Rendón et al. 2016; Freschi et al. 2019). However, to access and utilize all the information contained within genome sequences can be challenging (Marschall et al. 2018).

Here, to first gain more information on the structure of the global gene pool within our studied *P. aeruginosa* population, we identified its pan-genome. The pan-genome of 99 clinical isolates closely mirrored previously published *P. aeruginosa* pan-genomes (Mathee et al. 2008; Klockgether et al. 2011; Hilker et al. 2015; Valot et al. 2015). Of note, *P. aeruginosa* represents predominantly PAO1 and PA14 phylogroups (>98% to date). Recently, two additional taxonomy outliers with the PA7 phylogroup were identified (Freschi et al. 2019). We then used the pan-genomic information to describe genetic variation on a single-nucleotide level in a gene-wise approach. We identified a large number of *P. aeruginosa* core genes (3,814 genes) and aligned the orthologous sequences of all individual core genes. Any sequence variation at any position within the aligned genes gave information on SNPs at high accuracy.

In the last decade, MLST has been used to study the molecular epidemiology of bacterial pathogens (Maiden et al. 1998). MLST measures genetic variation in a limited set of housekeeping genes that are usually polymerase chain reaction amplified and sequenced. Sequencing results are then used to assign individual isolates of a population to STs according to their unique allele profiles. Today, with next-generation sequencing technologies, information across the entire genome can be obtained and STs can be identified at a much higher degree of discrimination. Therefore, discrimination power increases with the number of core genes and the quality of the ascertained sequence variations (Maiden et al. 2013).

Detailed knowledge on the full pattern of sequence variations among the core genes of the 99 clinical *P. aeruginosa* isolates paved the way for categorizing SNPs that define the branches of a phylogenetic tree (divergent SNPs), and SNPs that have been acquired independently in separate lineages, and not through inheritance from a common ancestor (convergent SNPs). We identified nearly 47.47% of the total SNPs as phylogroup dependent (divergent), whereas 21.38% were commonly found independently of the strain background (convergent). Approximately, 31.15% of the total SNPs were singleton SNPs with an average of 492 singletons per isolate. A large fraction of these singletons were identified in hypermutator strains, which exhibited a high mutation frequency due to mutations in DNA repair systems. For example, CH3570, CH2922, MHH15286, CH2824, CH2804, and CH2677 harbored >2,000 singleton SNPs, which is more than 4-fold than the average number of singletons per isolate. However, also both PA14 and PAO1 contained an above average numbers of singletons—811 and 1,118, respectively. This indicates that the two lab strains have deviated quite substantially from natural *P. aeruginosa* isolates. The categorization of SNPs that define the branches of a phylogenetic tree (divergent SNPs) in the 99 clinical *P. aeruginosa* isolates across the entirety of the core genes provided the basis for unprecedented detail into the phylogenetic relatedness between the individual isolates of the *P. aeruginosa* population.

It has been shown previously that the core genes are highly conserved (Dötsch et al. 2010). In accordance, the mean sequence diversity at the SNP level in this study was calculated to be 0.04 for the strict core genes. We further classified the convergent SNPs (independent on the strain background) into synonymous (80%) and nonsynonymous (20%) mutations. We determined the ratio of the nonsynonymous substitution rate (d*N*) to the synonymous substitutions rate (d*S*), d*N*/d*S* for the 3,814 core genes to be 0.14. The application of d*N*/d*S* methods can be challenging when analyzing within-species data, which represent mutations not yet fixed in the population (Rocha et al. 2006; Kryazhimskiy and Plotkin 2008). Nevertheless, to avoid a random high ratio, we used of a gene-wide approach on genes of 100% identical length across 99 isolates, which indicated that the *P. aeruginosa* core genome is, for the most part, not subject to adaptive evolution.

Previous comparative genomic studies have demonstrated that the core genes of bacteria play important roles in niche adaptation and virulence, especially in *P. aeruginosa* (Spencer et al. 2003; Wolfgang et al. 2003). Although many virulence determinants are generally a part of the core genome, novel accessory genomic sequences will continue to be detected (Wiehlmann et al. 2007; Silby et al. 2011; Pohl et al. 2014), and it has been suggested that the content of the accessory genome in *P. aeruginosa* determines environmental adaptability such as niche expansion (Lee et al. 2006; Mathee et al. 2008; Freschi et al. 2019). A recent study further revealed that intergenic mutations are more likely to be positively selected than coding mutations, especially as this enables essential genes to become targets of evolution in *P. aeruginosa* (Khademi et al. 2019). Furthermore, it seems that mutations in master regulators disproportionally impact the bacterial phenotype. The transcriptional profiles of some completely unrelated genotypes have been shown to exhibit similar phenotypes, and highly similar genotypes exhibit substantially different transcriptional phenotypes, mainly due to inactivating mutations in global regulators (Dötsch et al. 2015). Because inactivated global regulators often harbor indels or partial gene losses, many were not categorized in our pan-genome reconstruction as strict core genes.

In conclusion, we have shown that the core genome of the 99 clinical *P. aeruginosa* isolates analyzed in this study is conserved and is not subject to positive selection. The future analysis of full genomes that include soft-core, accessory, and intergenic regions will allow for more detailed information on the structure and dynamics of the *P. aeruginosa* SNP profile and how this contributes to bacterial phenotypes.

## Supplementary Material

Supplementary data are available at *Genome Biology and Evolution* online.

## Supplementary Material

evaa059_Supplementary_Data

## Notes

Data deposition: This project has been deposited at NCBI/SRA under the accession SRP160898.
