# Supplementary material for: Single-Nucleotide Polymorphism-Based Genetic Diversity Analysis of Clinical Pseudomonas aeruginosa Isolates
Source: Genome Biol Evol. 2020 Apr 1;12(4):396–406. doi: 10.1093/gbe/evaa059 (PMC7197496; doi:10.1093/gbe/evaa059)
Supplement: evaa059_Supplementary_Data [file evaa059_supplementary_data.zip › Supporting information captions.docx]

**Supporting information captions**

**Fig S1: Distribution of the core and soft-core genes in the group of 101 *P. aeruginosa* isolates.**

In addition to the 3,814 core genes present in 100% of genomes, 1,257 soft core genes are identified in 99% (626), 98% (286), 97% (175), 96% (118) and 95% (52) of the genomes respectively.

**Fig S2: The phylogenetic distribution of 99 clinical isolates and 52 fully sequenced public reference genomes of *P. aeruginosa.***

The current collection of clinical isolates is broadly distributed and comparable to the phylogenetic diversity of 52 previously sequenced *P. aeruginosa* genomes (as on 2016). Star and circle symbols represent clinical isolates and reference genomes respectively. The neighbor-joining tree shows three major groups PA14, PAO1 and PA7. PA7 strain type is a known outlier of the species. The phylogenetic distribution of our 99 clinical isolates is found to be comparable to the phylogenetic diversity of 52 previously sequenced *P. aeruginosa* strains.

**Fig S3: An example of SNP identification in the core genes visualized.**

As an example, few of the PA14_00010 (*dnaA*) core gene positions are visualized across the clinical isolates. SNPs are highlighted at different color scale, from grey to black. Position wise nucleotide diversity across clinical isolates are visualized and stored in the Bactome database (<https://bactome.helmholtz-hzi.de>).

**Fig S4: Phylogenetic tree based on all SNPs versus divergent SNPs.**

An approximate maximum-likelihood phylogenetic tree is constructed using fasttree and midpoint rooted (a) The tree is based on all SNPs (159,609) from 3,814 core genes. (b) The tree is based on only divergent SNPs (75,765) from 3,755 core genes. There is no significant differences between the two trees, however the tree based on divergent SNPs shows slightest variation in distances across and within the phylo-groups and depicts clear branches within the phylo-groups.

**Fig S5: dN/dS ratio (omega values) for genes present in 2-5 genomes.**

The mean pairwise dN/dS ratio for the genes present in 2-5 isolates (4,362 soft singletons) is determined to be 0.14.

**S1 File: Supplementary information file.**
