# Supplementary figures and images for: Single-Nucleotide Polymorphism-Based Genetic Diversity Analysis of Clinical Pseudomonas aeruginosa Isolates
Source: Genome Biol Evol. 2020 Apr 1;12(4):396–406. doi: 10.1093/gbe/evaa059 (PMC7197496; doi:10.1093/gbe/evaa059)

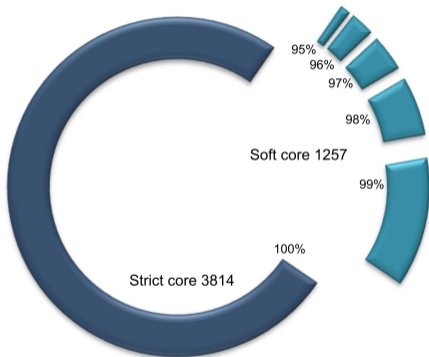

Supplement: evaa059_Supplementary_Data [file evaa059_supplementary_data.zip › FigS1.pdf]

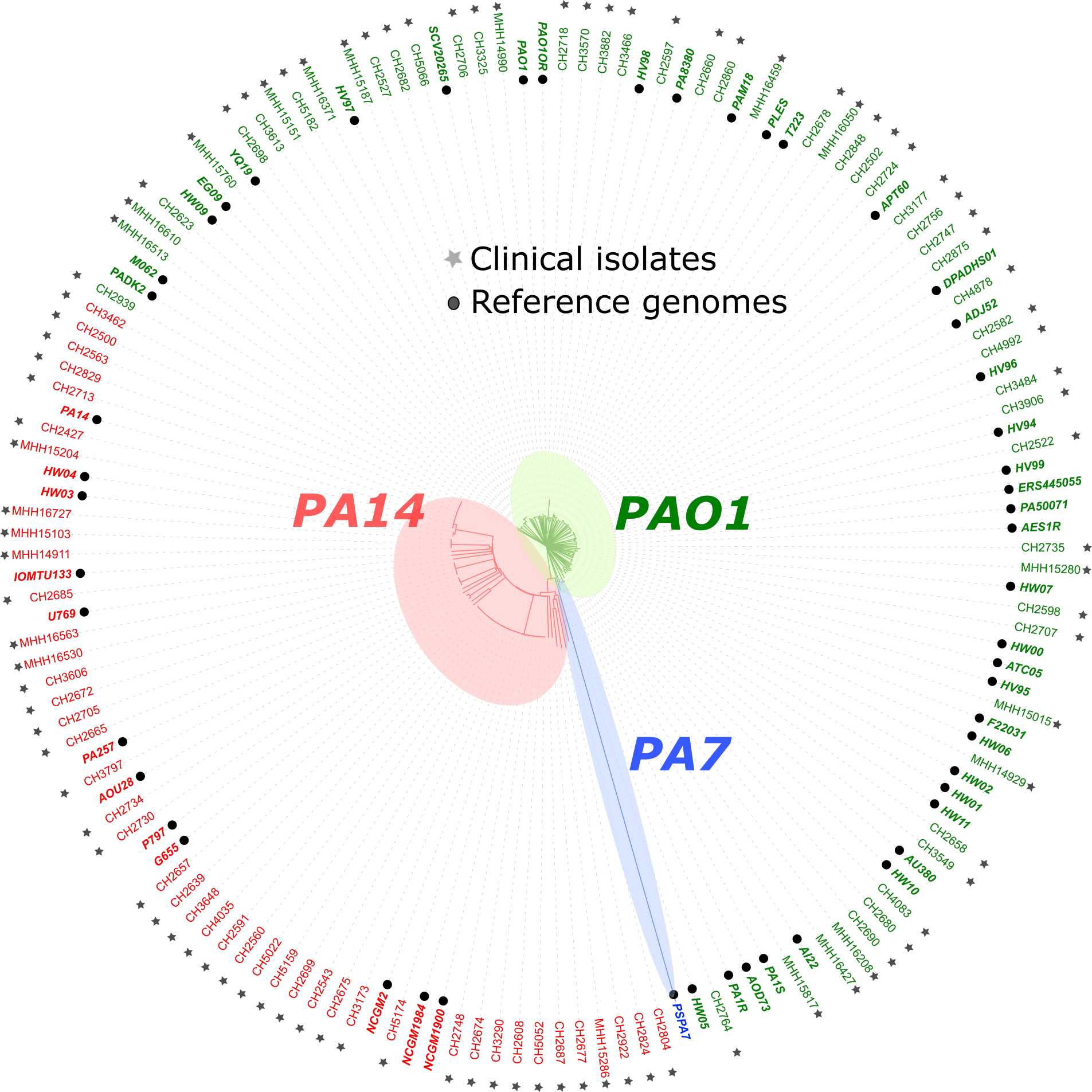

Supplement: evaa059_Supplementary_Data [file evaa059_supplementary_data.zip › FigS2.pdf]

I

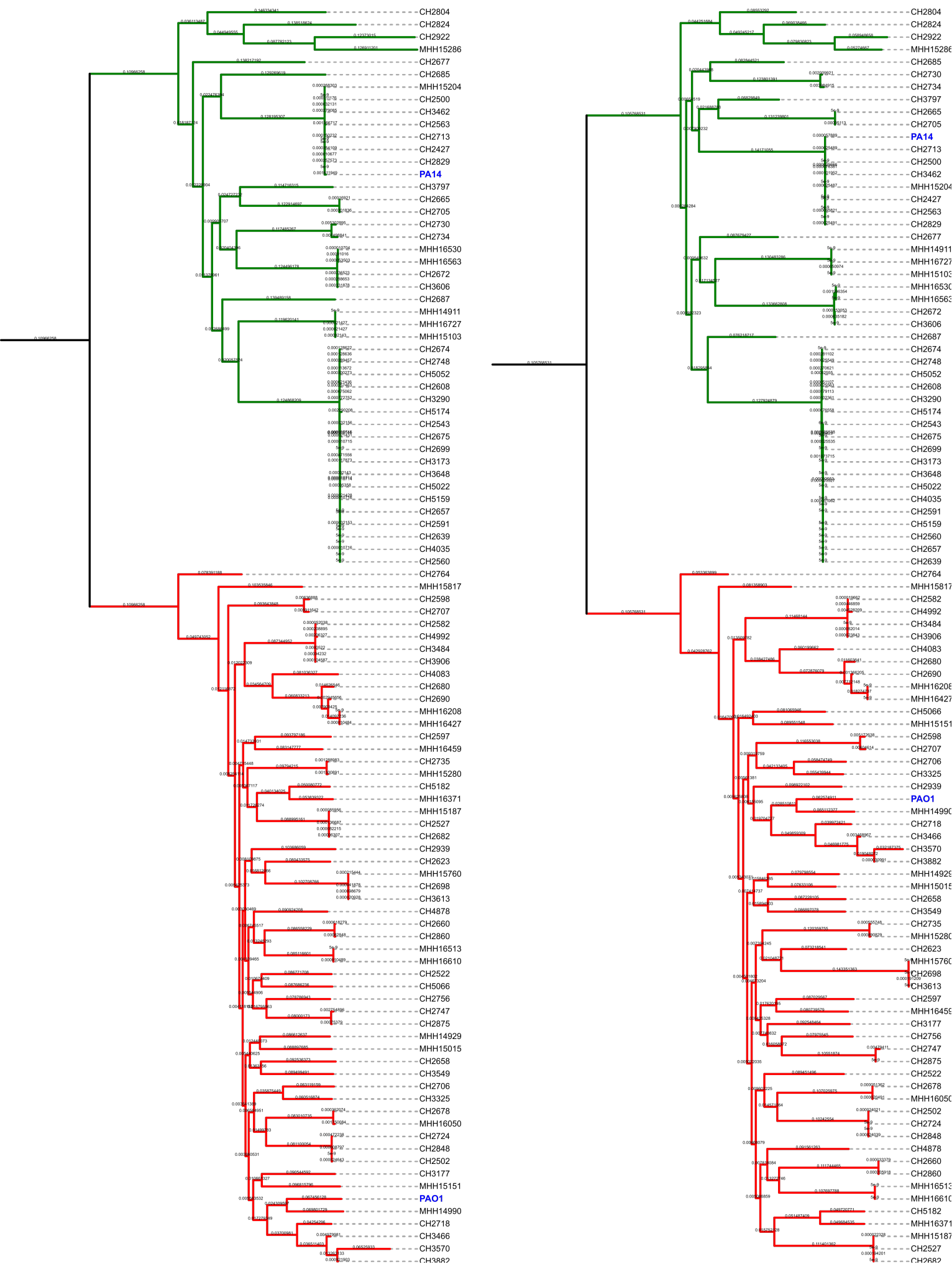

Supplement: evaa059_Supplementary_Data [file evaa059_supplementary_data.zip › FigS4.pdf]

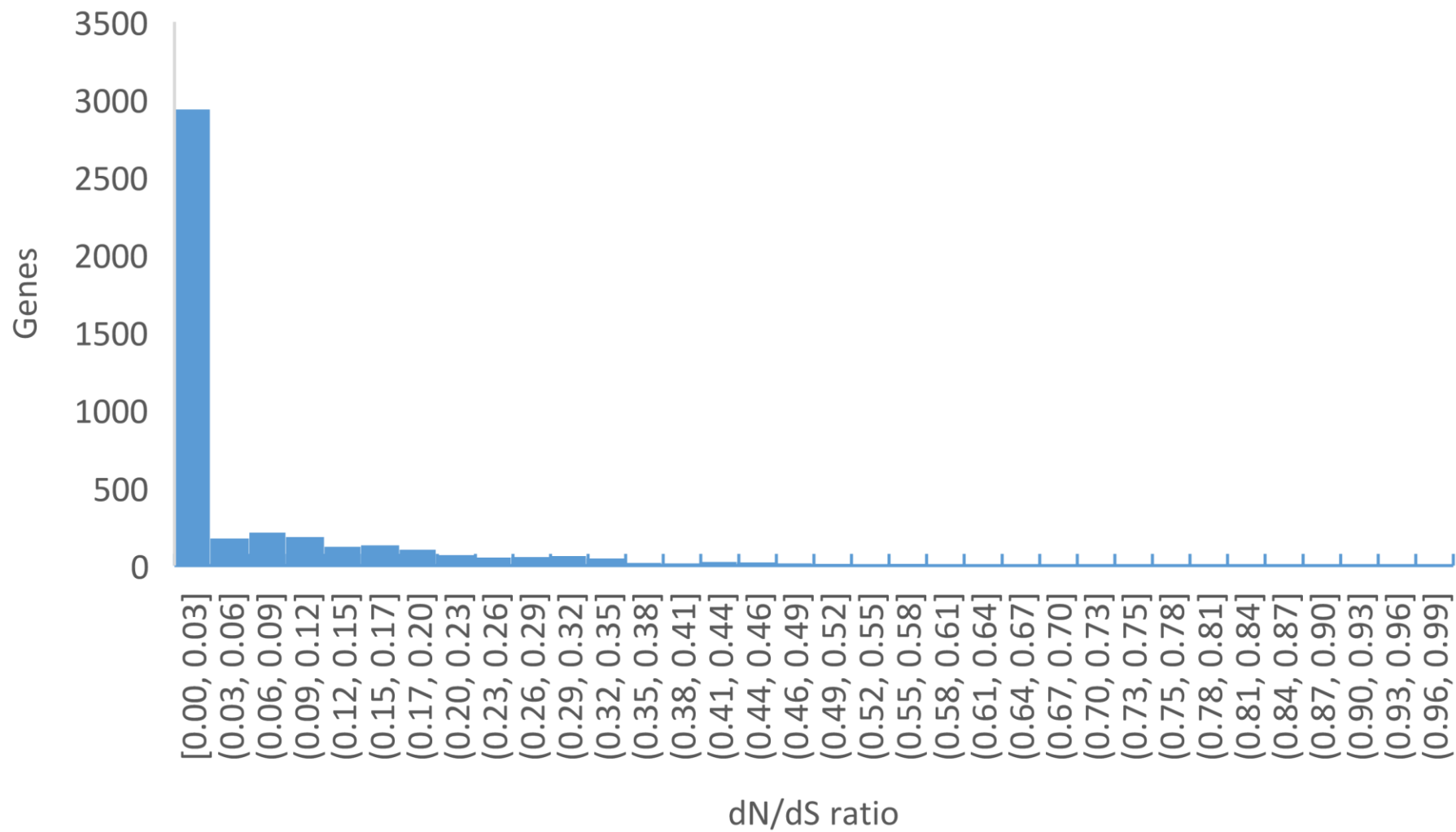

Supplement: evaa059_Supplementary_Data [file evaa059_supplementary_data.zip › FigS5.pdf]
